# Are Personality-Based Intellectual Styles Culture Specific or Universal?

**DOI:** 10.3389/fpsyg.2021.717670

**Published:** 2021-10-20

**Authors:** Li-fang Zhang

**Affiliations:** Faculty of Education, The University of Hong Kong, Hong Kong, Hong Kong SAR, China

**Keywords:** Jungian personality styles, career personality styles, culture specific, universality, cultural stereotypes

## Abstract

Traditionally, it had been commonly believed that individuals in the same culture have personalities distinct from those of individuals in other cultures. This article examines this belief by critically reviewing relevant literature generated from two of the most widely investigated personality-based style constructs in the field of intellectual styles: the Jungian personality styles and the career personality styles proposed by Holland. It aims at answering the question of whether personality-based intellectual styles are culture specific or they are universal. To achieve this aim, based on the two broad cultural systems derived from Hofstede's model of four cultural dimensions and two major style types from Zhang and Sternberg's threefold model of intellectual styles, two research hypotheses were made. To test the hypotheses, two types of empirical literature centered on each of the two personality-based styles are reviewed: (1) cross-cultural comparative studies; and (2) within-culture studies investigating the association of the two style constructs with other human attributes and outcomes. Results suggest that although personality-based styles are related to culture, they cannot be culture specific; rather, they are fundamentally universal. These findings carry scientific value and have practical implications for education and beyond.

One of the eternal scholarly pursuits in the field of psychology is to investigate the cultural specificity and universality of personalities (Heine and Buchtel, 2009). Such a sustained research endeavor among scholars is chiefly motivated by the fact that stereotypical views about the personalities of people from different cultures continue to be strongly held by many. In this era of globalization and with the world's heightened focus on cultural awareness, deepening our understanding of the said issue has become more important than ever before. The present article investigates the cultural specificity and universality of personality-based intellectual styles by testing two hypotheses guided by the two broad cultural systems derived from Hofstede's (1980) theory of four cultural dimensions and further by two types of styles based on Zhang and Sternberg (2005) threefold model of intellectual styles. In general, it was hypothesized that in cultural contexts characterized by low power distance, low uncertainty-avoidance, individualism, and masculinity (Hofstede, 1980), people would be more likely to use creativity-generating personality-based intellectual styles; Furthermore, the use of such styles would be more likely to be associated with better attributes and outcomes. It was also hypothesized that in cultural contexts characterized by high power distance, high uncertainty-avoidance, collectivism, and femininity, people would be more likely to use norm-favoring styles; Moreover, the use of such styles would be more conducive to better attributes and outcomes (see specific hypotheses under “Personality-based Styles and the Four Cultural Dimensions: Conceptual Links and Hypotheses”).

Intellectual styles, an all-encompassing term for such constructs as cognitive styles, learning styles, teaching styles, personality-based styles, and thinking styles, refer to people's preferred ways of processing information and dealing with tasks (Zhang and Sternberg, 2005). In their threefold model of intellectual styles, Zhang and Sternberg (2005) classified all individual styles in the existing style constructs into three types: Type I, Type II, and Type III styles. Type I styles are more creativity-generating and they denote higher levels of cognitive complexity. This type of styles can be considered to be more adaptive because they are routinely associated with more desirable human attributes and outcomes such as higher levels of cognitive development, creativity, and open mindedness. Type II styles suggest a norm-favoring tendency, and they denote lower levels of cognitive complexity. This type of styles can be considered maladaptive because they are more often related to undesirable human attributes and outcomes such as lower levels of identity development, surface learning approach, and rigidity. Type III styles may show the characteristics of either Type I or Type II styles, depending on the stylistic demands of specific situations or tasks. The adaptivity of Type III styles is variable because the ways in which this type of styles are related to other human attributes and outcomes have been largely inconsistent (see also Zhang, 2017).

Like personalities and abilities, styles are significantly associated with human learning and performance in different cultural contexts. However, styles are neither personalities nor abilities; but rather, they are at the interface between personalities and abilities (Sternberg, 1997). Styles can be activity-centered, cognition-centered, and personality-centered (Grigorenko and Sternberg, 1995).

This article focuses on the two most widely researched personality-based intellectual styles: Jung's (1923) construct of personality styles (also known as personality types) and Holland's (1973) construct of career personality styles (also known as career interest types). The key question to be answered is: Are personality-based intellectual styles culture specific, or are they universal?

Before explaining what is meant to be universal or culture specific in terms of personality-based intellectual styles, it should be acknowledged that the concept of universality, or precisely, psychological universality, is a complex phenomenon (e.g., Norenzayan and Heine, 2005; Van de Vijver and Leung, 2021). Indeed, based on a comprehensive review of the literature, Norenzayan and Heine (2005) identified four levels of universality: accessibility, functional, existential, and non-universal.

In the context of this article, universality (and cultural specificity, for that matter) lies in its functionality. Specifically, personality-based intellectual styles are considered universal if the same styles can be identified and function (i.e., the manner in which styles are associated with other human attributes and outcomes) in the same way in different cultural systems. By contrast, personality-based styles are deemed culture specific if particular styles are found to be consistently pervasive in some cultural contexts, but not in others (i.e., non-universal); and if the same types of styles function systematically differently in different cultural systems (see under the heading “Personality-based Intellectual Styles and Hofstede's Model of Four Cultural Dimensions” for details regarding style types and cultural systems).

It is argued that although personality-based intellectual styles are influenced by culture, they cannot be culture specific. Instead, personality-based intellectual styles are fundamentally universal. To substantiate this argument, the remainder of this article is divided into four parts. The first part introduces the two personality-based style constructs and the primary measure for each construct; highlights Hofstede's (1980) four cultural dimensions; establishes the conceptual link between culture and the two personality-based style constructs; specifies the two research hypotheses concerning the relationships between culture and intellectual styles; and describes the method for selecting the relevant literature. To test the first hypothesis, the second part of this article presents research evidence from cross-cultural comparative studies of each of the style constructs. To test the second hypothesis, the third part reviews studies on the association of each of the two style constructs with other human attributes and outcomes. The fourth and final part makes concluding remarks.

## Personality-Based Intellectual Styles and Hofstede's Model of Four Cultural Dimensions

### Personality-Based Intellectual Styles and Key Measurements

Of the many intellectual style constructs documented in the literature (e.g., Zhang and Sternberg, 2005), two personality-based style constructs have been most widely investigated in different cultural contexts. These are Jung's (1923) construct of personality styles, and Holland (1973) construct of career personality styles.

#### Jungian Personality Style and the Myers-Briggs Type Indicator

Jung (1923) contended that individuals have a propensity for attending selectively to elements in a learning environment, looking for learning environments compatible with their reported personality types (or styles; the term “personality styles” is used hereafter to align with the literature on intellectual styles), and shying away from incompatible ones. According to Jung, these psychological preferences fall along three dimensions: extroversion-introversion, sensing-intuitive, and thinking-feeling. Myers and McCaulley (1985) extended Jung's classification by adding a fourth dimension—judging-perceiving. *Extraverted* (E) individuals tend to be oriented toward the outer world of actions, objects, and people, whereas *introverted* (I) individuals prefer the inner world of concepts and ideas. *Sensing* (S) individuals prefer to seek the fullest possible experience of what is immediate and real, whereas *intuitive* (N) individuals tend to seek the broadest view of what is insightful and possible. *Thinking* (T) individuals tend to make decisions on the basis of logical and rational planning, whereas *feeling* (F) individuals have an inclination for making decisions based on harmony among subjective values. *Judging* (J) individuals have a predisposition to seek closure, at times without adequate exploratory activities, whereas *perceiving* (P) individuals tend to be attuned to incoming information and open to new events and changes until they have to make a decision. Based on the three-fold model of intellectual styles (Zhang and Sternberg, 2005), the perceiving and intuitive personality styles are Type I styles; the judging and sensing personality styles are Type II styles; and thinking, feeling, introversion, and extraversion are Type III personality styles (see also Zhang, 2017 for empirical evidence).

The *Myers-Briggs Type Indicator* (MBTI), first published in 1943 (Myers and Briggs, 1943) and at present in its 19th print (Myers et al., 1998), a forced-choice self-administered test, is the inventory most frequently used to assess the four dimensions of preferences. The various versions of the MBTI have been translated into different languages and administered in different cultural contexts; they have proven to possess good psychometric properties (see Myers et al., 1998; Zhang, 2013).

Jung (1958) argued that psychological preferences can be manifested not only among individuals but also among civilizations, cultures, nationalities. Indeed, the four underlying personality dimensions as assessed by the MBTI have been found in different cultural contexts.

#### Career Personality Style and the Self-Directed Search

According to Holland (1973), people can be classified into six personality types corresponding to six occupational environments: realistic, investigative, artistic, social, enterprising, and conventional. *Realistic* individuals like to work with things and enjoy out-door activities but may lack social skills. *Investigative* individuals like to be engaged in scientific work but often lack leadership skills. *Artistic* individuals are inclined to deal with tasks that provide them with opportunities to utilize their imagination but often lack clerical skills. *Social* individuals prefer to work in situations in which they can interact and collaborate with others but may lack mechanical and scientific skills. *Enterprising* individuals, like *social* individuals, prefer to work in environments in which they can interact with others but, unlike *social* individuals, they enjoy taking on leadership roles in their collaborative endeavors. Finally, *conventional* individuals prefer to work on data in well-structured situations but often lack artistic skills. According to the threefold model of intellectual styles (Zhang and Sternberg, 2005), the artistic and investigative career personality types are Type I career personality styles; the conventional and realistic career personality styles are Type II career personality styles; and the enterprising and social career personality styles are Type III styles (see also Zhang, 2017 for empirical evidence).

First published in 1971 (with the latest version published in 1994), the *Self-Directed Search* (SDS, Holland, 1994) is the most popular inventory used to assess the six career personality styles. The SDS is a self-administered and self-scored inventory in which the respondents indicate their likes and dislikes of the activities and occupations in the six types of career environments and rate their competencies in each of the six areas. The SDS has been translated into more than 30 languages and has generated thousands of empirical studies all over the world (e.g., Swan, 2005). The great majority of these studies resulted in satisfactory reliability and validity data. The *SDS Manual* (Holland, 1994) reported good internal consistency (using KR-20) and test-retest reliability data as well as good concurrent and predictive validity data.

To overcome the gender bias for which the SDS is often criticized, Zhang (1999) designed the *Short-version Self-directed Search* (SVSDS). The SVSDS is a self-report questionnaire containing 24 items, with each set of four items contributing to the assessment of one of the six career personality styles. Reliability and validity data of the SVSDS were recorded in a number of publications (e.g., Zhang, 2001; Ng, 2015).

### Culture and Hofstede's Model of Four Cultural Dimensions

#### Culture

Various insightful definitions of culture (e.g., Tylor, 1958; Adler, 2001) have been proposed. In this article, Hofstede's (1990) definition of culture— “the collective programming of the mind that distinguishes the members of one category of people from another” (p.4), is adopted. This article restricts its survey of cross-cultural studies of personality-based intellectual styles to cultural distinctions as a function of jurisdictions^1^ and ethnic groups within a jurisdiction.

#### Hofstede's Model of Four Cultural Dimensions

In the latter half of the Twentieth Century, several theoretical models on culture were put forward by scholars in different academic fields, including anthropology (Hall, 1976), psychology (Markus and Kitayama, 1991), and sociology (Berry, 1991). Relatively more recently, Yamagishi et al. (2008) analyzed the culture-bound nature of human behaviors from the game-theoretic perspective. However, Hofstede's (1980) model of four cultural dimensions constructed based on his investigation in the field of management has been selected to guide the present discussion for its conceptual links with the two personality-based intellectual style constructs (see under “Personality-based Styles and the Four Cultural Dimensions: Conceptual Links and Hypotheses”). In the data gathered from 40 countries, Hofstede (1980) identified four basic cultural dimensions: Power distance, uncertainty avoidance, individualism (vs. collectivism), and masculinity (vs. femininity).

Power distance concerns human inequality. It refers to the extent to which the less powerful members of a society accept the unequal distribution of power and expect this to be the case. The level of power distance is socially determined and is endorsed by both the followers and the leaders. A low power distance society is creativity-generating because it allows individuals more freedom, whereas a high power-distance society tends to stifle creativity because a much stronger emphasis is put on conformance, hierarchies, and rules (Jones and Herbert, 2000).

Uncertainty avoidance pertains to a society's tolerance for ambiguity. People in low uncertainty-avoidance societies are likely to be more tolerant of novel ideas and are less rule-oriented. In contrast, people in high uncertainty-avoidance societies tend to be less tolerant of novel ideas and to seek clarity through rules and regulations. People from higher uncertainty-avoidance societies may reduce uncertainty by relying on guidance of other people as opposed to thinking for themselves, whereas people from low uncertainty-avoidance societies are more likely to be reflective and to think for themselves.

Individualism vs. collectivism concerns the relationship between the individual and the collectivity in a given society. This relationship does not merely refer to people's ways of living together (e.g., in families); but also, “it is intimately linked with societal norms” (Hofstede, 1980, p. 214). This means that this relationship influences individuals' “mental programming” (Hofstede, 1980, p. 214). Individualist societies are more tolerant of individual thoughts and behaviors. For this reason, individuals in such societies are less concerned with doing “safe” things and are more risk-taking. By contrast, collectivist societies are less tolerant of individual thoughts and behaviors, which makes individuals more concerned about doing things in ways that are accepted by other members of the society through avoiding risk-taking.

Masculinity vs. Femininity refers to the distribution of emotional roles between males and females. The predominant socialization patterns are for males to be more assertive and for females to be more nurturing. The stability of gender-role patterns has more to do with socialization than with biological factors (Hofstede, 1980). Assertiveness and decisiveness are more valued in masculine societies, whereas rule-following and obedience are more valued in feminine societies. It follows that people from masculine societies tend to be engaged in new ways of thinking, whereas people from feminine societies tend to be engaged in more conventional thinking (Hofstede, 1980, 1990).

Hofstede's conceptualization of the four cultural dimensions has gained strong empirical support. By the year 2001, Hofstede had constructed an index for each of the four cultural dimensions for 66 jurisdictions. Despite some exceptions, a general trend emerged. That is, the economically more developed jurisdictions normally fall on one end of each of the four continua: low power distance (L_PD_), low uncertainty avoidance (L_UA_), individualism (I), and masculinity (M)—referred to as “L_PD_L_UA_IM” hereafter; while the economically less developed jurisdictions usually fall on the other end of each of the four continua: high power distance (H_PD_), high uncertainty avoidance (H_UA_), collectivism (C), and femininity (F)—referred to as “H_PD_H_UA_CF” hereafter.

### Personality-Based Styles and the Four Cultural Dimensions: Conceptual Links and Hypotheses

Observant readers must have noticed the strong resemblance between the characteristics of Type I personality-based intellectual styles and those of Hofstede's L_PD_L_UA_IM societies, despite the fact the former represent individual characteristics and the latter, societal ones. Similarly, one could hardly fail in noticing the correspondence between the characteristics of Type II styles and those of H_PD_H_UA_CF societies.

On the basis of the conceptual similarities between intellectual styles and Hofstede's cultural dimensions, one should expect that people in Hofstede's L_PD_L_UA_IM jurisdictions and ethnic groups be more likely to use Type I personality-based intellectual styles, and that people in Hofstede's H_PD_H_UA_CF jurisdictions and ethnic groups be more likely to use Type II styles (*Hypothesis 1*)^2^. One should further anticipate that in L_PD_L_UA_IM jurisdictions or ethnic groups, Type I personality-based intellectual styles serve individuals better in that a more frequent use Type I personality-based intellectual styles would be related to more adaptive attributes and better outcomes. By contrast, in H_PD_H_UA_CF jurisdictions and ethnic groups, Type II personality-based styles would serve individuals better in that a more frequent use of Type II personality-based styles would be associated with more adaptive attributes and better outcomes (*Hypothesis 2*). In the next section, the method of selecting the literature for testing these hypotheses is described.

### Literature Selection Method

For a study to be included in this review, its research must, first and foremost, involve one of the two personality-based intellectual style constructs: the Jung personality styles and career personality styles. Furthermore, the study has to be one of the following two types of empirical investigations. The first type concerns cross-cultural comparison—either direct or indirect comparison. Direct comparative studies refer to those involving actual comparison of measurement scores of research participants from two or more cultural groups; while indirect comparative studies refer to those conducted independently within a cultural group (i.e., either a jurisdiction or an ethnic/racial group), but with the patterns of their findings compared across studies. The second type of studies investigated the association of either of the two personality-based intellectual style constructs with other human attributes and outcomes.

In what follows, the above mentioned two types of studies are introduced. Cross-cultural comparative studies will be presented first, followed by research on personality-based intellectual styles and their outcomes.

## Cross-Cultural Comparative Research on Personality-Based Intellectual Styles^3^

To what extent can the hypothesis that people from L_PD_L_UA_IM jurisdictions and ethnic groups (i.e., cultures) would tend to use Type I styles and that people from H_PD_H_UA_CF cultures would tend to use Type II styles be confirmed? What do these findings say about the cultural specificity and universality of the two personality-based style constructs? In answering these questions, this part reviews cross-cultural comparative studies on the Jungian personality styles (Jung, 1923) and career personality styles (Holland, 1973).

### Jungian Personality Styles: Cross-Cultural Comparative Studies

Based on Zhang and Sternberg (2005) classification of intellectual styles and the specifications of Hofstede's cultural dimensions, individuals from societies that fall on the L_PD_L_UA_IM ends of Hofstede's cultural continua would be more intuitive and perceiving (i.e., scoring higher on these two Type I personality styles), whereas people from societies that fall on the H_PD_H_UA_CF ends would be more sensing and judging (i.e., scoring higher on these two Type II styles)^4^. Although this prediction has been confirmed by findings in the majority of studies (e.g., Hedegard and Brown, 1969; Levy et al., 1972; Hammer and Mitchell, 1996; Broer and McCarley, 1999), it has also been challenged by those in a number of studies (e.g., Shade, 1983, 1986; Tobacyk and Cieslicka, 2000).

The majority of the studies supporting the prediction have been conducted at the within-jurisdiction level. The primary interest of the researchers of these studies was to identify the predominant Jungian personality styles of their research participants from different ethnic groups. In 1969, Hedegard and Brown found that, compared with their Caucasian counterparts, students of African descent exhibited a preference for using more tangible ways (i.e., more sensing) than intellectual ways (i.e., less intuitive) in dealing with their environments. Likewise, Levy et al. (1972) identified significantly higher proportions of judging and sensing types among university students of African descent than among students of European descent. In a national sample (1,267 adults aged 18–94 years) ethnically matched in proportion to the 1990 census in the United States, Hammer and Mitchell (1996) found a significantly higher proportion of sensing types among African Americans in comparison with the general sample highly dominated by European Americans.^3^,^4^

Findings confirming the hypothesis have also been obtained at a broader cultural level. For instance, research on the Jungian personality styles among mainland Chinese business administrators and professionals (e.g., Yao, 1993; Broer and McCarley, 1999) revealed that the Type II sensing and judging styles were prevalent. In the same vein, Huang and Huang (1991) found an overrepresentation of sensing and judging styles among Taiwanese university students.

Nevertheless, the first hypothesis has also been challenged by empirical findings that were either statistically insignificant or directly opposed to the hypothesis. For example, given the economic and social disadvantages that African Americans have commonly experienced in comparison with their European-American counterparts in the United States, one should expect African Americans to score higher on the judging and sensing personality styles and European Americans to score higher on the perceiving and intuitive personality styles, on average. Nevertheless, according to Shade (1986) review of the literature, no significant difference was found in the MBTI styles of people of African descent in comparison with those of European descent before school grade 3 or after the first year of college. Furthermore, contrary to the hypothesis, Shade (1983, 1986) empirical research on ninth-grade students consistently found that students of African descent were generally more perceiving, whereas European Americans were more judging.

#### Summary

In this section, the existing cross-cultural comparative studies involving the two Type I and two Type II Jungian personality styles have been highlighted. Although the existing literature is pretty outdated, with the most recent study having been conducted in the year 2000, the research findings have well-addressed the first hypothesis. The hypothesis has surely been supported by the majority of the empirical studies. Nevertheless, it has also been challenged by some of the studies. That is, compared with individuals in the H_PD_H_UA_CF cultural systems, those in the L_PD_L_UA_IM ones did score higher on the Type I intuitive and perceiving personality styles more frequently; however, the reverse has also been found—at a level that was higher than statistical chance. Given the mixed findings, one should say that although culture does matter significantly in people's Jungian personality styles, they are not culture specific.

### Career Personality Styles: Cross-Cultural Comparative Studies

Typically, cross-cultural studies of career personality styles based on Holland's (1973) model have one of the following three objectives: (1) to identify different patterns of career personality styles among different cultural groups; (2) to examine the criterion-related validity of the *Self-Directed Search* (SDS); and (3) to test the underlying structure (i.e., structural fit) of the career personality styles of racial-ethnic groups within jurisdictions (Zhang, 2013).

Patterns of career personality styles refer to the ways in which test respondents' scores on the aforementioned six scales (i.e., realistic, investigative, artistic, social, enterprising, and conventional) are ranked. Based on the classification of the threefold model of intellectual styles (Zhang and Sternberg, 2005) and further founded on the characteristics of the four cultural dimensions (Hofstede, 1990), it was anticipated that individuals from societies that fall on the L_PD_L_UA_IM ends of Hofstede's cultural continua would be more likely to score higher on the Type I artistic and investigative career personality styles (i.e., express stronger interest in these two types of career environments), whereas individuals from societies that fall on the H_PD_H_UA_CF ends would be more likely to score higher on the Type II conventional and realistic career personality styles^5^.

Criterion-related validity concerns both concurrent validity (i.e., how well the test takers' SDS results correspond to their current jobs or academic majors) and predictive validity (i.e., the degree to which the test takers' SDS results are consistent with their career aspirations). According to Hofstede's (1980) model, the H_PD_H_UA_CF societies would inevitably put constraints on individuals' career personality styles, including depriving individuals of the opportunities to be exposed to certain types of occupations and to develop the career personality styles they might have developed had they been socialized in a L_PD_L_UA_IM culture. Following this logic, one would be on solid grounds for anticipating that such constraints would bring about poorer prediction of people's career personality styles and poorer Holland's model fit for individuals in H_PD_H_UA_CF cultures.

Structural fit refers to how well the SDS data obtained from test respondents fit Holland's model. For the same reason just mentioned with respect to criterion-related validity, one would expect that data from L_PD_L_UA_IM cultures show better fit with Holland's model than do those from H_PD_H_UA_CF cultures.

Given the popularity and long history of Holland's (1973) theory and the use of the SDS, research testing the above predictions has not been as fruitful as one would expect. Support and challenges for the above mentioned predictions are, nevertheless, informative with respect to the cultural specificity and universality of career personality styles.

#### Patterns: Empirical Evidence

Regarding the anticipation on the patterns of career personality styles among people of different cultural contexts, only two studies have been identified. In a first study, Gade et al. (1984) compared the career personality styles (as assessed by the SDS) between Native American high school students from two Indian tribes—Swampy Cree students, who were boarding students in an all-white community school district, and Peguis students, who were enrolled in a local reserve school district. The Swampy Cree female students scored significantly higher on the Type I investigative personality style, whereas the Peguis males scored significantly higher on the Type II conventional style. Irrespective of the fact that significant difference was not found across genders between the two tribal groups, the difference identified may suggest that there might have been an acculturation effect related to students' being exposed to the culture in the all-white community on their career personality styles. That is, the Swampy Cree students' displaying more of the characteristics of the investigative career personality style could partially be attributed to their interaction with white students. In other words, although individuals' career personality styles certainly have a great deal to do with culture, one cannot claim that people from different cultures possess distinct career personality styles that are static. Instead, career personality styles are dynamic—with the necessary stimuli, they can be developed in individuals of any cultural context. This suggests that career personality styles cannot be culture specific.

Results from a more recent study (Morris, 2016) communicated mixed messages regarding the cultural aspect of career personality styles. After analyzing data gathered (between 2005 and 2014) from over one million residents of different ethnic groups in the U.S., Morris (2016) concluded that “although generally very small” (p.612), some differences in career personality styles have been found. For example, in comparison with those who did not indicate ethnicity, Asians, Indians, and Middle easterners tended to score higher on the investigative personality style. It can be contended that this finding supported the anticipation regarding the patterns of career personality styles because in the United States, the Asians, Indians, and Middle easterners tend to be economically better off compared with other ethnic minority groups. Such an economic advantage might have provided individuals from these groups with opportunities to be exposed to environments in which their Type I investigative career personality style was developed. Nevertheless, contrary to the anticipation, Blacks and Native Americans, despite being two of the most economically disadvantaged ethnic groups in the United States, scored significantly lower on the Type II realistic career personality style. Therefore, once again, the cultural specificity of career personality styles was not established.

#### Criterion-Related Validity: Empirical Evidence

Two criterion-related studies were identified and both lent support to the anticipation that the SDS's predictive validity would be relatively lower for individuals from Hofstede's H_PD_H_UA_CF cultures. Khan et al. (1990) found that Pakistani university students' SDS codes were not good predictors for their career readiness. Likewise, Leung and Hou (2001) found that compared with the predictive validity reported in previous studies conducted in the United States, the correspondence between the Hong Kong Chinese high school students' SDS high-point career interest codes and their tentative choices of university majors and careers was generally lower.

The question that arises is: Do the above hypothesis-supporting findings indicate that career personality styles are culture specific? The answer is negative. The lower predictive validities could have been attributable to educational systems (assuming that educational systems are part of cultural practices) that tend to exercise more power and control, placing constraints on students' development of career personality styles. Take the study by Leung and Hou (2001) as an example, Hong Kong students were, at the time when the study was conducted, required to choose either a science stream or an arts stream at the end of junior high school (i.e., 9th grade in the United States). Such early and often blind commitment to an area of study might have prevented students from developing Type I career personality styles. One of the major objectives of the 2012 school (and university) curricular reform in Hong Kong was to broaden students' career personality styles. If Leung and Hou's (2001) study is replicated in Hong Kong today, the predictive validity of the SDS for the same population will likely to be improved. Put differently, career personality styles are not unique to people of particular cultures. With necessary conditions, career personality styles can be developed within individuals from any cultural context. In fact, abundant empirical evidence for the malleability of intellectual styles, including that of personality-based styles, has been documented (Zhang, 2013).

#### Structural Fit: Empirical Evidence

It was anticipated that the SDS data from L_PD_L_UA_IM cultures would have a better structural fit with Holland's model than those from H_PD_H_UA_CF cultures. Findings concerning this anticipation have been equivocal. For example, Einarsdòttir et al. (2002) found that the underlying structure of career personality styles of university students in Iceland (an L_PD_L_UA_IM jurisdiction) resembled that of U.S. benchmark samples. The researchers of the study attributed this resemblance chiefly to the fact that the Icelandic culture and the U.S. culture held similar rankings on Hofstede's cultural dimensions and that both jurisdictions are noted for a high level of economic prosperity.

Incorporating the notion of economic development (as evaluated by gross domestic product per capita; GDPPC) and two of the four Hofstede's cultural dimensions (masculinity-femininity and individualism-collectivism), Rounds and Tracey (1996) conducted a meta-analysis of data sets from 76 international samples (representing 18 jurisdictions), 20 ethnic samples in the United States, as well as 73 benchmark samples in the United States. Although the degree of data fit with Holland's model was not significantly associated with masculinity-femininity and GDPPC, better model fit was achieved for jurisdictions with more individualistic values than for those with more collective values.

Nonetheless, the conjecture concerning Holland's model fit with respect to Hofstede's cultural system continua was also challenged. For example, within the U.S., Swanson (1992) concluded that the structure of career personality styles among African-American university students resembled that of European-American university students. Likewise, at the level of nations/jurisdictions, contrary to the anticipation, data from the Australian and Canadian samples (individuals from L_PD_L_UA_IM cultures) demonstrated a significantly poorer model fit in comparison with the U.S. benchmark data. These findings clearly disputed the argument for the cultural specificity of career personality styles.

Cross-nationally, holding gender and occupation constant, Fouad and Dancer (1992) identified strikingly similar structures of career personality styles among engineers in Mexico and in the United States. Similar research findings had been obtained as early as the 1960s. For example, Lonner (1968) concluded that American, German, Swiss, and Austrian psychologists were more similar to one another than to accountants within their respective countries. Such empirical evidence is indicative that career personality styles could be universal.

#### Summary

In this section, each of the three conjectures (i.e., patterns, predictive validity, and structural fit) has been empirically examined through reviewing cross-cultural comparative research on career personality styles. Although both support and challenges have been found for the research hypothesis tested, it is fair to state that the amount of challenges outweighed that of support. Given this and the dynamic nature of career personality styles (Iliescu et al., 2013; Zhang, 2013), one should say that although individuals' career personality styles can be significantly affected by culture, they cannot be culture specific.

## Personality-Based Intellectual Styles and Their Outcomes

The second hypothesis in this article states that Type I personality-based intellectual styles would serve individuals better in L_PD_L_UA_IM cultural systems and that Type II styles would serve individuals better in H_PD_H_UA_CF cultural systems. To what extent has this hypothesis been empirically supported? What does the literature say about the nature of personality-based styles in terms of their cultural specificity and universality? This part addresses these questions by reviewing research on the association of the two personality-based style constructs with diverse human attributes and outcomes.

In her monograph “The Value of Intellectual Styles,” Zhang (2017) critically reviewed studies concerning the relationship of a wide range of intellectual style constructs (including the Jungian personality styles and career personality styles) with various human attributes and outcomes. Therefore, those studies will not be recapitulated here; instead, they are briefly introduced in the first section of this part. In the second section, studies beyond Zhang's (2017) work will be examined in greater detail.

### Studies in Zhang's Review

Within the context of elucidating the value of intellectual styles (i.e., whether or not some styles carry more adaptive values than do others), Zhang (2017) analyzed studies investigating the relationship of each of the two personality-based styles with other human attributes and outcomes. In terms of the studies centered on the personality styles defined by Jung (1923), 54 studies involving 20 other attributes and outcomes were reviewed. Some examples of these attributes and outcomes are character strengths, creativity, creative and critical thinking, leadership behaviors and practices, leadership styles, organizational seniority, personality traits, teaching excellence, teaching performance, and tendency for embracing new teaching technology. Spanning more than five decades, these studies were conducted among students, teachers, and personnel in the workplace in seven jurisdictions, including Canada, Finland, France, South Africa, Taiwan, the United Kingdom, and the United States (see the Appendix for more details on each study).

As shown by the findings presented in the Appendix, with the exception of seven studies that yielded results that either were statistically non-significant or partially (or fully) disconfirmed the hypothesis that in L_PD_L_UA_IM cultures, Type I styles would better serve individuals, the remaining 47 studies indicated that the Type I Jungian personality styles (i.e., intuitive and perceiving) were conducive to desirable attributes and outcomes, regardless of cultural contexts. Interestingly, the seven exceptional results suggesting that Type II styles served individuals better did not occur in the studies conducted in South Africa and Taiwan (H_PD_H_UA_CF societies); but rather, they were all obtained in studies carried out in L_PD_L_UA_IM cultures (i.e., six in the United States and one in Finland). Certainly, one could argue that because the majority of the studies shown in the Appendix were conducted in the U.S., it is reasonable that these exceptional results occurred in studies conducted in the U.S. Statistically, such an argument is sound. Be that as it may, these results did show that Type II personality styles were occasionally associated with desirable outcomes in L_PD_L_UA_IM cultures; at the same time, Type I personality styles were proven equally desirable in H_PD_H_UA_CF cultural contexts such as South Africa and Taiwan. Furthermore, given that the great majority (i.e., 47) of the 54 studies suggested that Type I personality styles served individuals better in terms of their being associated with desirable attributes and outcomes in all seven jurisdictions and due to the absence of evidence showing that Type II personality styles served individuals better in H_PD_H_UA_CF cultural systems, the Jungian personality styles can only be deemed fundamentally universal, not culture specific.

In the same book, Zhang (2017) reviewed 14 studies that tested individuals' career personality styles (Holland, 1973) against five other attributes and outcomes: achievement motivation, the big three and the big five personality traits, creativity, educational satisfaction, and job satisfaction. Spanning more than four decades, these studies were conducted among students, teachers, and working adults in five jurisdictions: Australia, Belgium, Hong Kong, mainland China, and the United States (see also the Appendix for more details on each study).

With no exception, all of the 14 studies suggested that Type I career personality styles (i.e., artistic and investigative) were related to adaptive attributes and outcomes and that Type II styles (i.e., conventional and realistic) were related to maladaptive attributes and outcomes. This finding fully disconfirmed the prediction that Type II styles would serve individuals better in H_PD_H_UA_CF cultural systems such as in Hong Kong and mainland China. Thus, these studies suggested that career personality styles are universal, not culture specific.

### Studies Beyond Zhang's Review

In order to verify if the above conclusion would hold true in studies outside Zhang's (2017) review, a thorough search of the literature (published between 2012 and March 2021) was conducted. As expected, both Jung's (1923) conceptualization of personality styles and Holland's (1973) model on career personality styles continued to be productive in generating empirical work. However, only 12 studies are relevant to the topic of this article – nine centered on the Jungian personality styles and three on career personality styles. Other studies are not suitable for being examined here because they either only focused on the Type III^6^ Jungian introversion-extraversion personality styles (e.g., Al-Dujaily et al., 2013; Murphy et al., 2017) or dealt with the relationships of personality styles with attributes that are not obviously value laden (e.g., investment behaviors in Insler et al., 2016; whistleblowing in Park et al., 2014).

#### Studies Centered on the Jungian Personality Styles

Of the nine studies centered on the Jungian personality styles, four involved cognitive outcomes (Karimnia and Mahjubi, 2013; Kim et al., 2013; Ayoubi and Ustwani, 2014; Rashid and Duys, 2015), two concerned affective outcomes (Ahmed, 2015; Choi et al., 2018), one pertained to ego development (Vincent et al., 2013), and two concerned interpersonal behaviors (Brandt and Laiho, 2013; Furnham and Crump, 2014). In the following, these studies are introduced.

##### Cognitive Outcomes With the Jungian Personality Styles

A first study was conducted among 35 Iranian university students majoring in translation (Karimnia and Mahjubi, 2013). The participants took a 72-item version of the *Myers-Briggs Type Indicator* (MBTI) and were evaluated on the quality of their translation (from English to Persian) of three short paragraphs conceptualized within Reiss (1971) text typology—operative, informative, and expressive. Results showed that although students' personality styles did not make a significant difference in their performance on translating operative and informative texts, students classified as higher on the intuitive personality style (a Type I style) significantly outperformed their sensing (Type II style) counterparts in translating expressive text.

The superiority of the intuitive personality style over the sensing style has also been demonstrated in Ayoubi and Ustwani (2014) research among 89 university students in Syria. The researchers examined the relationship between students' scores on an Arabic version of the MBTI (From M) and their grade point averages (GPAs). As asserted by the researchers, the most critical conclusion drawn from this study was that intuitive students had significantly higher GPAs than did their sensing peers.

The positive association of the intuitive personality style with better cognitive performance has also been revealed in Kim et al. (2013) study of 85 third-year computer science university students in New Zealand. The participants took the MBTI (version not specified) and responded to 40 questions (20 on declarative knowledge and 20 on procedural knowledge) after learning and discussing all of material learned on computer. Although students did not differ in their performance on declarative knowledge as a function of personal styles, those classified as intuitive performed significantly better on procedural knowledge than did those classified as sensing.

Finally, in the study conducted among 74 students pursuing their Master's degree in counseling in the United States (Rashid and Duys, 2015), the Type I perceiving personality style showed superiority over the Type II judging personality style. The participants took the MBTI (Myers et al., 1998) and the *Role Category Questionnaire* (RCQ; Burleson and Waltman, 1988) measuring cognitive complexity in counselor trainees. Results suggested that higher scores on the RCQ were positively related to the perceiving personality style, but negatively related to the sensing style.

##### Affective Outcomes and the Jungian Personality Styles

Both studies concerning the association between affective outcomes and personality styles were carried out in Asia. In the first study (Ahmed, 2015), 130 postgraduate students in business management in India responded to the MBTI (Myers et al., 1998) and the *Resilience Inventory* (Guttman, 1999). Results revealed that compared with sensing students, intuitive students were significantly more resilient in that they demonstrated stronger abilities to either bounce back or thrive when faced with perpetual uncertainty and chaos.

Conducted in South Korea, the second study (Choi et al., 2018) addressed the relationship between emotional intelligence and the Jungian personality styles. Participants were 72 long-term practitioners of mind-body training (MBT) and a comparative group of 62 healthy individuals. Form G of the MBTI and a Korean version of the *Saehan Media EQ Test* (Moon, 1999) were used to assess the two aforementioned constructs. Although no statistically significant relationship was found between personality styles and emotional intelligence in the comparative group, a significantly positive relationship was identified between the intuitive personality style and emotional intelligence in the MBT group.

##### Ego Development and the Jungian Personality Styles

Ego development, individuals' growth in ways of constructing meaning throughout the lifespan (Loevinger, 1976), has long been recognized as one of the most comprehensive constructs in developmental psychology (Westenberg and Block, 1993). Its positive development unfolds along the hierarchy of achieving greater self and interpersonal awareness; decreasing defensiveness and increasing flexibility; becoming more reflective, more skilled in interacting with the environment, more tolerant of differences and ambiguity; increasing cognitive complexity; and achieving a stronger sense of responsibility and personal autonomy (Cook-Greuter, 1999).

Although only one study (Vincent et al., 2013) has investigated the relationship between ego development and the Jungian personality styles, its findings aligned well with the anticipation that Type I personality styles would be related to adaptive attributes. The study was conducted among 374 adults participating in 11 community leadership development and two professional development programs in Australia. Participants responded to the MBTI (Form M) and the *Washington University Sentence Completion Test* (Hy and Loevinger, 1996). Results indicated that the intuitive personality style was significantly related not only to higher levels of ego development on program entry but also to greater ego development in the process of the programs.

##### Interpersonal Behaviors and the Jungian Personality Styles

The term “interpersonal behaviors” is adopted here as a broad concept to refer to two specific attributes: (1) transformational leadership behivors; and (2) interpersonal dysfunctional behaviors. Each of the attributes has been tested against the Jungian personality styles. The first study (Brandt and Laiho, 2013) was conducted among 459 leaders and 378 subordinates working in various sectors in Finland. The leaders took the Finnish research “F-version” of the MBTI (Myers et al., 1998) and evaluated themselves on transformational leadership as measured by the *Leadership Practice Inventory* (LPI; Posner and Kouzes, 1988); while the subordinates evaluated their leaders on the LPI. Intuitive male leaders and perceiving leaders (both male and female) rated themselves as more challenging (a key dimension of transformational leadership) than did their sensing and judging counterparts. Moreover, the subordinators also considered male leaders with stronger perceiving personality style as more challenging. Together, these results suggested that the Type I intuitive and perceiving styles were positively associated with practicing the transformational leadership style—a leadership style that has long been proven to be adaptive in various cultural contexts (Majauskaite and Alonderiene, 2015).

In the second study, Furnham and Crump (2014) investigated the relationship between personality styles and dysfunctional interpersonal behaviors among 4,812 British working adults. The participants responded to the MBTI-Form G (Briggs and Myers, 1987) and the *Hogan Development Survey* (Hogan and Hogan, 1997). Results showed that the participants classified as judging (a Type II style) scored significantly higher on five of the 11 dysfunctional interpersonal behaviors.

##### Summary

In this section, nine studies investigating the Jungian personality styles with four categories of human attributes and outcomes were reviewed. Although each of the studies was conducted in a different jurisdiction (Australia, Finland, India, Iran, New Zealand, South Korea, Syria, the United Kingdom, and the United States), the results from all of these studies, like the studies reviewed in Zhang's (2017) book, pointed to one direction—that is, the Type I intuitive and perceiving personality styles were invariantly positively related to the more desirable attributes and outcomes involved in the studies; while the Type II sensing and judging styles were consistently negatively associated with desirable attributes and outcomes—irrespective of cultural contexts. That is to say, these studies outside Zhang's (2017) review also have disconfirmed the anticipation that Type II styles would better serve individuals in H_PD_H_UA_CF cultural systems. Once again, it could be contended that the Jungian personality styles are universal, not culture specific.

### Studies Centered on Career Personality Styles

As noted earlier, the literature search conducted for the purpose of writing this article merely secured three relevant studies beyond Zhang's (2017) review. Nevertheless, the research findings are informative vis-à-vis the hypothesis on how each of the two types of career personality styles would be related to human attributes and outcomes in Hofstede's (1990) two broad types of cultural systems.

In the first study, Littman-Ovadia et al. (2014) investigated the relationship between career personality styles and mindfulness among 156 full-time employees in Israel. Mindfulness in this context refers to the tendency for being open-minded to novelty whereby the individual actively makes cognitive classifications and distinctions (Langer, 1978). The participants took the Hebrew version of the occupations section of the SDS (Holland, 1985) and the 14-item *Langer Mindfulness Scale* (Pirson et al., 2012). Results suggested that the two Type I career personality styles (i.e., artistic and investigative) as well as the Type II realistic style were significantly related to mindfulness. This finding is in line with Zhang's (2015) notion of successful intellectual styles—in that mindfulness requires both Type I and Type II styles, especially the former.

In the second study, Ding et al. (2015) examined the relationship between career personality styles and performance on the *Graduate Record Examination* (GRE) among 106 graduate students majoring in school counseling and mental health counseling in the United States. The SDS-Form R (Holland, 1994) was used for assessing the participants' career personality styles. Results indicated that the Type I investigative career personality style statistically predicted students' scores on all sections of the GRE.

In the third and final study, Pellerone et al. (2015) tested the association of career personality styles with school performance, identity development, and school absences among 417 senior secondary school students in Italy. The participants' career personality styles were assessed with the SDS, and their identity development was evaluated by the *Ego Identity Process Questionnaire* (Balistreri et al., 1995). It was found that the Type I investigative personality style was positively significantly related to students' performance in all four subject areas (i.e., human performance, scientific performance, language performance, and technical performance). By contrast, the Type II realistic personality style was negatively correlated with students' performance in all four subject areas, while the Type II conventional style was negatively related to human performance. Furthermore, the artistic career personality style was positively associated with adaptive identity statuses (i.e., exploration and achievement), but negatively with diffusion—a maladaptive identity status. In addition, while the investigative career personality style was negatively related to school absences, the realistic style was positively so.

#### Summary

In this brief section, three studies, each conducted in a different jurisdiction, were introduced. Each study examined the relationship of career personality styles (Holland, 1973) to a different attribute or outcome. Across the three studies, the Type I artistic and investigative styles were positively related to adaptive attributes and outcomes, but negatively with maladaptive ones; whereas the Type II conventional and realistic styles were negatively related to students' performance. Interestingly, it was in the study of participants from Israel, an economically advanced (and an increasingly more individualistic society since the 1960s) jurisdiction that the Type II realistic style was found to make a positive contribution to mindfulness (Littman-Ovadia et al., 2014). Thus, like the studies centered on the Jungian personality styles, studies on the career personality styles disconfirmed the prediction that Type II styles would better serve individuals in Hofstede's (1990) H_PD_H_UA_CF cultural systems. Such a dispute against the said hypothesis suggests, once again, that career personality styles cannot culture specific.

## Closing Remarks: Conclusions, Limitations, Future Directions, Scientific Significance, and Practical Implications

The aim of this article was to ascertain whether the Jungian personality styles and career personality styles are culture specific or universal. Two hypotheses made based on Hofstede's (1990) model of four cultural systems and Zhang and Sternberg (2005) threefold model of intellectual styles were tested with relevant empirical work. This part draws conclusions on the basis of the findings presented in the preceding two parts; critiques the existing research on the relationship between culture and the two personality-based intellectual style constructs, pointing out its limitations and possible future research directions; explains the scientific value of the present review; and discusses the practical implications of the key findings for education and beyond.

### Conclusions

Cross-cultural comparative studies yielded largely mixed results, with some confirming the first hypothesis (i.e., individuals from L_PD_L_UA_IM cultural systems would be more likely to adopt Type I personality-based styles, while individuals from H_PD_H_UA_CF cultural systems would be more likely to adopt Type II styles) and others disconfirming it. Such mixed findings suggest that personality-based styles cannot be culture specific for the simple reason that it has not been empirically established that individuals from one broad cultural system consistently scored higher on particular types of personality-based styles than did those from the other.

At the same time, the universality of personality-based intellectual styles has been strongly revealed by the empirical findings challenging the second general hypothesis of this article. Unlike hypothesized, regardless of the cultural contexts in which the empirical studies were conducted, Type I personality-based intellectual styles invariably served the research participants better in that individuals scoring higher on these styles tended to display more desirable attributes and achieve better outcomes, whereas Type II styles generally served people poorly. Furthermore, on several exceptional occasions when Type II personality-based styles did serve individuals better, those exceptions occurred in L_PD_L_UA_IM cultures, not in H_PD_H_UA_CF ones.

Consequently, considering the two bodies of the literature collectively, one should say that although culture certainly plays a crucial role in personality-based intellectual styles, none of the styles is unique to, or “owned” by, any culture. As a matter of fact, personality-based intellectual styles have long been proven to be accessible to people in different cultural contexts and similar patterns of Jungian personality styles (e.g., ESTJ—extraversion-sensing-thinking-judging personality style) and those of career personality styles (e.g., IAS—investigative-artistic-social career personality style) have been ascertained in different cultural contexts (e.g., Holland, 1994; Myers et al., 1998). Culture is dynamic, and so are personality-based intellectual styles (Zhang, 2013). Based on both the existing literature and the present findings, and further founded on the dynamic nature of personality-based styles, one must conclude: personality-based intellectual styles cannot be culture specific; but rather, they are principally universal.

Indeed, the personality-based intellectual styles cannot be culture specific for four additional reasons—at the very least. First, jurisdiction/ethnic group is not the only dimension along which each of the cultural-dimension indices (i.e., power distance, uncertainty avoidance, individualism vs. collectivism, and masculinity vs. femininity) varies. The indices also vary as a function of other socialization variables, most evidently, age, gender, academic discipline, educational level, and occupation (Hofstede, 1980). Second, within each jurisdiction/ethnic group, individuals of different social classes and, of course, people of the same social class, may fall on different points along each of the four cultural-dimension continua. Third, with temporal evolution and the increasingly faster speed of modernization (as commonly seen in economic growth—except for some periods such as the current era of the COVID-19 pandemic), and in this highly globalized world, those cultures that once tended toward, say, collectivism, might begin to manifest more individualism (Matsumoto, 2002; Zhang, 2013). Finally, individuals of the same cultural system may exhibit quite different characteristics in relation to Hofstede's cultural dimensions. For instance, the Japanese culture is usually featured by its avoidance of conflict and of overt criticism at the individual level. However, at the group or organizational level, uncertainty is often well-acknowledged (Westwood and Low, 2003). In fact, it was with these caveats that the two general hypotheses regarding the relationship between Hofstede's (1980) two broad cultural systems and Zhang and Sternberg (2005) Type I and II personality-based intellectual styles were put forward.

### Limitations and Future Research Directions

Obviously, very few studies concerning the second hypothesis (i.e., the one concerning how the two different types of personality-based intellectual styles would serve individuals differently in each of the two broad cultural systems) have been conducted in H_PD_H_UA_CF cultural systems. Thus, conclusions drawn here might cast doubt in the minds of some readers. However, it should be remembered that there is also variability among jurisdictions within each the two broad cultural systems (i.e., H_PD_H_UA_CF and L_PD_L_UA_IM)—with respect to both styles and positions along each of Hofstede's (1980) four cultural-dimension continua. As such, the conclusions drawn from the existing findings obtained from research participants from various cultural contexts (despite being predominantly L_PD_L_UA_IM ones) should ease the minds of individuals who may be less confident in the present conclusions. Nevertheless, future researchers are encouraged to conduct more empirical studies in H_PD_H_UA_CF cultural contexts, particularly studies that test the association of personality-based intellectual styles with human attributes and outcomes.

### Scientific Significance

Guided by the model of four cultural dimensions (Hofstede, 1980) and the threefold model of intellectual styles (Zhang and Sternberg, 2005), this article pioneered the examination of the link between culture and personality-based intellectual styles. The present findings carry scientific value.

Traditionally, in trying to understand cultural differences in personality (or, in any other human attributes or outcomes, for that matter), researchers tended to target directly at identifying differences as a function of nation/jurisdiction and racial/ethnic groups. Results from these between-group comparative studies, despite playing an important role in understanding people's differences in personality, may have unintended negative consequences. For example, according to the representativeness heuristic (Bordalo et al., 2016), between-group comparisons are likely to perpetuate stereotypes. Precisely, group differences found and reported in publications often mislead receivers of such information to form stereotypical views about groups investigated (Bordalo et al., 2016; Quinn, 2020). The present finding that the cultural specificity of personality-based intellectual styles cannot be established echoes previous scholars' call for cautioning against stereotypes (Bordalo et al., 2016; Quinn, 2020). Researchers who are engaged in between-group cross-cultural comparative studies are reminded to be prudent in presenting, interpreting, and generalizing their findings.

Furthermore, the present finding that Type I personality-based styles are desirable in virtually all jurisdictions and racial/ethnic groups suggests that in studying cultural differences in personality-based styles, researchers should go far beyond engaging in between-group comparisons. They are advised to examine how personality-based intellectual styles are related to other human attributes and outcomes within each cultural context and to ascertain meaningful patterns and commonalities of relevant relationships across cultural contexts. In this way, the nature of personality-based intellectual styles in relation to culture can be better understood.

Finally, the evidence-based conclusion that personality-based intellectual styles are fundamentally universal reinforces the long-standing argument for the value-laden nature of intellectual styles (Kogan, 1989; Renzulli and Sullivan, 2009, Zhang, 2017). That is, largely irrespective of cultural contexts, some styles are regarded more desirable than are others. In the same vein, the present conclusion highlights the argument for the malleability of intellectual styles (Henson and Borthwick, 1984; Sternberg, 1997; Zhang, 2013). Personality-based intellectual styles are accessible to people in virtually all cultural contexts. With appropriate stimuli, desired intellectual styles can be fostered in all cultural contexts. From Yamagishi's et al. (2008) game-theoretic perspective, behaviors that are newly developed in particular cultures would be interpreted as “strategies adapted to a set of collectively created social incentives” (p. 579). However, researchers in the field of intellectual styles (e.g., Witkin, 1962; Sternberg, 1997; Zhang, 2017) would argue that such development manifests the beginning of a process for preferences to be formulated. Having been a passionate researcher in the field of intellectual styles and with the abundant empirical support (e.g., Zhang, 2013, 2017), the present author strongly endorses the latter view.

### Practical Implications

Apart from being scientifically significant, the present findings also have two practical implications for education and beyond. The first is derived from the finding that people from H_PD_H_UA_CF cultural systems do not necessarily use less creativity-generating personality-based intellectual styles than do people from L_PD_L_UA_IM ones. With this knowledge, while working with students, educational practitioners should not only take students' cultural backgrounds into consideration, but also be vigilant against forming or holding stereotypical views about students from any culture. The same should apply to the general public when interacting with people from different cultural contexts.

The second practical implication is enlightened by the key finding that, regardless of cultural contexts, Type I personality-based intellectual styles serve individuals far better than do Type II styles. With this knowledge, individuals from all cultures should be more conscious of cultivating Type I styles—both within themselves and among others (see Zhang and Sternberg, 2020).

## Contribution to the Field

Grounded in the conceptual link between the two broad cultural systems informed by Hofstede's model of four cultural dimensions and those of Type I and Type II intellectual styles proposed by Zhang and Sternberg, two research hypotheses were made and tested. Although some crosscultural comparative studies suggested cultural differences as hypothesized, others either failed in identifying any significant cultural difference in the personality-based styles or sustained differences challenging the hypothesis. Meanwhile, within-culture studies consistently showed the desirability of creativity-generating personality-based intellectual styles in terms of their association with adaptive attributes and outcomes-regardless of cultural contexts. Supporting the conclusion that personality-based intellectual styles are fundamentally universal, these findings offer a new lens through which researchers could investigate the nature of personalities in relation to culture. Meanwhile, these hard data should remind us all, people in and outside the education arena, of guarding against cultural stereotypes about personality styles.

## Data Availability Statement

The original contributions presented in the study are included in the article/Supplementary Material, further inquiries can be directed to the corresponding author/s.

## Author Contributions

The author confirms being the sole contributor of this work and has approved it for publication.

## Funding

This research was funded by the Seed Fund for Basic Research as administered by the University of Hong Kong, Hong Kong Special Administrative Region, the People's Republic of China.

## Conflict of Interest

The author declares that the research was conducted in the absence of any commercial or financial relationships that could be construed as a potential conflict of interest.

## Publisher's Note

All claims expressed in this article are solely those of the authors and do not necessarily represent those of their affiliated organizations, or those of the publisher, the editors and the reviewers. Any product that may be evaluated in this article, or claim that may be made by its manufacturer, is not guaranteed or endorsed by the publisher.

## Footnotes

^1^The term “jurisdiction” is used here to refer to both a country and a special administrative region of a particular country. For example, as a special administrative region of the People's Republic of China, Hong Kong is referred to as a jurisdiction, as are other countries.

^2^Hypothesis 1 is derived from a more general hypothesis on the relationships between culture and intellectual styles in the work by myself and Sternberg, titled “Culture and Intellectual styles,” published in *Handbook of Intellectual Styles* (Zhang et al., 2012).

^3^This part of the literature review is largely drawn on a chapter by myself and Sternberg, titled “Culture and Intellectual styles,” published in *Handbook of Intellectual Styles* (Zhang et al., 2012).

^4^In line with the conceptual links between the two broad cultural systems (i.e., L_PD_L_UA_IM and H_PD_H_UA_CF) and Zhang and Sternberg's threefold model of intellectual styles, only the two Type I and two Type II Jungian personality styles are pertinent to the two general hypotheses in this article.

^5^In line with the conceptual links between the two broad cultural systems (i.e., L_PD_L_UA_IM and H_PD_H_UA_CF) and Zhang and Sternberg's threefold model of intellectual styles, only the two Type I and two Type II career personality styles are pertinent to the two general hypotheses in this article.

^6^Recall that the two key hypotheses in this article only concern Type I and Type II personality-based intellectual styles.

## Appendix: Studies on Culture and Personality-based Intellectual Styles in Zhang (2017)

**Table:**

**Jungian Personality Styles (measured with MBTI) with Other Attributes and Outcomes (see under “Construct”)**					
**Author (year)**	**Construct**	**Measure assessing the construct**	**Sample description**	**Jurisdiction**	**Key findings**
^PU^Barrett (1991)	Congenial classroom environment	The Stern Classroom Environment Index (Stern, 1971)	34 vocational teachers and their 622 students	the United States	P^+^; J^−^ teachers S+; N+ teachers; but S teachers as more than N teachers, unexpectedly
^Non−sig.^Brown and Reilly (2009)	Transformational leadership	the Multifactor Leadership Questionnaire (Bass and Avolio, 1990)	408 leaders and 2,411 followers	the United States	Leaders' self-ratings: N^+^ ; S^−^; Subordinates' ratings of their leaders: non-significant
Chambers et al. (2003)	Willingness to use educational technology	A 20-item questionnaire (Callister and Burbules, 1990)	164 novice teachers	the United States	N^+^; S^−^
Chenhall and Morris (1991)	Resource allocation decision-making behaviors	A brief case study of decisions on relevance of opportunity costs	64 middle- and senior-level managers	France	N managers were more thoughtful than S managers
Choong and Britton (2007)	Creativity	The Values in Action Inventory of Strengths (Peterson and Seligman, 2004)	98 adult volunteers	the United States	N^+^; S^−^
Dollinger et al. (2004)	Creativity	the Creativity Behavior Inventory (Hocevar, 1979); the Creative Personality Scale (Gough, 1979); and the Test for Creative Thinking - Drawing Production (Urban and Jellen, 1996)	94 undergraduate students	the United States	N^+^; S^−^
Furnham (1996)	Big 5 PTs	NEO-PI (Costa and McCrae, 1985)	160 middle to senior managers	the United Kingdom	N^+^, P^+^; S^−^, J^−^ with openness
Furnham et al. (2003)	Big 5 PTs	NEO-PI - Form S (Costa and McCrae, 1985)	900 British adults	the United Kingdom	N^+^; S^−^ with openness
Furnham et al. (2007)	Big 5 PTs	NEO-PI (Costa and McCrae, 1985)	over 3,000 managers	the United Kingdom	N^+^, P^+^; S^−^, J^−^ with openness
Furnham et al. (2009)	Creativity	The Consequences test (Christensen et al., 1953)	2603 middle and senior managers of multinational communication organizations	the United Kingdom	N^+^, P^+^; S^−^, J^−^
^FU^Gentry et al. (2007)	Managerial derailment	The observer-form of BENCHMARKS (Lombardo and McCauley, 1994)	6,124 managers	the United States	N^+^; P^+^
^PU^Hautala (2006)	Transformational leadership	the Leadership Practices Inventory (Posner and Kouzes, 1988)	439 leaders and 380 subordinates	Finland	Leaders' self-ratings: N^+^ and P^+^; [Subordinates' ratings of their leaders: S^+^ (unexpected)]
Hough and Ogilvie (2005)	Leaders' behaviors in a simulated strategic decision-making environment	Decisiveness and perceived effectiveness in responding to problems in “The Looking Glass Experience” (a week-long seminar)	749 experienced managers	the United States	N managers made most effective decisions; S managers made least effective decisions.
Houtz et al. (1994)	Creative thinking	The Torrance Test of Creative Thinking (Torrance, 1984)	46 pre-service teachers	the United States	N^+^, P^+^; S^−^, J^−^
Kagan and Smith (1988)	Teachers' classroom behaviors	Teacher Structure Checklist (Webster, 1972), records of the frequency of six types of verbal behaviors, and a classroom map	51 kindergarten teachers	the United States	N^+^, P^+^ positively with child-centered; S^+^, J^+^ negatively with teacher-centered
MacDonald et al. (1994)	Big 5 PTs	NEO-PI - Form S (Costa and McCrae, 1985)	209 university students	Canada	N^+^; S^−^ with openness
Mills (2003)	Teaching excellence	Award-winning versus non-award-winning	63 exemplary teachers and 1,128 middle school teachers	the United States	Compared with the normative teachers, the exemplary teachers were more intuitive but less sensing
^**1^ Moutafi et al. (2007)	Organizational seniority	Self-reported managerial level	900 adults	the United Kingdom	N^+^; S^−^
Munro et al. (2012)	Character strengths	The Values in Action Inventory of Strengths (Peterson and Seligman, 2004)	69 pre-service teachers	South Africa	N^+^; S^−^ with transcendence
Overbay et al. (2009)	Dispositional resistance to change	The Resistance to Change Scale (Oreg, 2003)	237 elementary and middle school teachers	the United States	N^−^; S^+^
Purcell and Wilcox (2007)	Satisfaction in using educational technology	A short reflection paper	56 pre-service teachers	the United States	N^+^; S^−^
Quenk (1966)	Optimism	Daydreams recorded over 10 days	57 adults	the United States	N^+^
Reid (1999)	Job Satisfaction	the Maslach Burnout Inventory - Form Ed (Maslach and Jackson, 1981)	189 female elementary school teachers	the United States	N^+^
Ross et al. (2005)	Dogmatism	The Troldahl-Powell Dogmatism Scale (1965)	422 female pre-service teachers	the United Kingdom	N^−^, P^−^; S^+^, J^+^
Rushton et al. (2006)	Teaching excellence Award-winning versus non-award-winning	39 school district-level Teacher of the Year (ToY) recipients;	993 school teachers	the United States	ToY recipients: N^+^, P^+^; Normative teachers: S^+^, J^+^
Rushton et al. (2007)	Teaching excellence	Award-winning versus non-award-winning	58 exemplary teachers; 993 school teachers	the United States	The exemplary teachers were more intuitive and perceiving than the two “normative” groups
^PU^Schmidt (1989)	Teaching behaviors	the researcher's live observation of a one-hour lesson of each participant	43 graduate associate instructors	the United States	N+; S^−^ with reinforcement, approval, and teacher modeling; J^+^; P- with reinforcement (unexpected)
Smith et al. (1995)	Willingness to use educational technology	A 20-item questionnaire (Callister and Burbules, 1990)	138 teachers	the United States	N^+^; S^−^
Srivastava et al. (2010)	Creativity	the Barron-Welsh Art Scale (Barron, 1963), the Adjective Checklist Creative Personality Scale (Gough, 1979), and the Torrance Tests of Creative Thinking - Figural and Verbal versions (Torrance, 1990)	32 bipolar disorder patients, 21 unipolar major depressive disorder patients, 22 creative controls, and 42 healthy controls	the United States	N^+^; S^−^
^Non−sig.^Vaughan and Knapp (1963)	Pessimism	25 items describing an optimistic and a pessimistic outlook	75 male undergraduates	the United States	Not statistically significant
Walla (1988)	Congenial classroom environment	Classroom Environment Index (long form, CEI-971) - students' evaluation of teachers	34 vocational teachers and 638 vocational students	the United States	N^+^, P^+^; S^−^, J^−^ teachers
^PU^Watson and Hillison's (1991)	Job Satisfaction	The Minnesota Satisfaction Questionnaire (Weiss et al., 1967)	63 teachers	the United States	S^−^-P^−^ (P^−^ unexpected)
Yang and Lin (2004)	Creativity	the Chopsticks Creativity Test (Wu, 1998)	1119 male students of a senior high school	Taiwan	N^+^; S^−^
Carless (1999)	Big 5 PTs	The NEO-PI-R (Costa and McCrae, 1992)	139 working adults and students	Australia	A^+^ with openness (for both genders); A^+^ with openness (for males)
Costa et al. (1984)	Big 3 Personality traits	The Neuroticism-Extraversion-Openness Inventory (Costa and McCrae, 1980)	394 adults	the United States	A^+^; C^−^ with openness (for both gemders) I^+^ with openness (for females)
De Fruyt and Mervielde (1997)	Big 5 PTs	The NEO-PI-R (Costa and McCrae, 1992)	934 university students	Belgium	A^+^; I^+^ with openness
Fu (2017)	Achievement motivation	The Achievement Motivations Measure - Revised (Elliot and Murayama, 2008)	282 university students	mainland China	I^+^ with performance approach; C^+^ with mastery avoidance and performance avoidance
Gade et al. (1988)	Educational satisfaction	Survey of Study Habits and Attitudes (Brown and Holtzman, 1967)	596 Native American school students	Canada	I^+^; R^−^
Gottfredson et al. (1993)	Big 5 PTs	The NEO-PI (Costa and McCrae, 1985)	725 Navy trainees	the United States	A^+^ with openness (for both males and females) I^+^ with openness (for females).
Holland et al. (1994)	Big 5 PTs	The NEO-PI (Costa and McCrae, 1989)	298 adults	the United States	A^+^; I^+^ with openness (both genders); I^−^ with neuroticism (males)
Kelly and Kneipp (2009)	Creativity	Scale of Creative Attributes and Behaviors (Kelly, 2004)	115 undergraduate students	the United States	A^+^ with all five creativity components. R^+^ with spontaneity
Larson and Borgen (2002)	Big 5 PTs	The NEO-PI-R (Costa and McCrae, 1992)	323 adolescents	the United States	A^+^; I^+^ with openness
Schinka et al. (1997)	Big 5 PTs	The NEO-PI-R (Costa and McCrae, 1992)	1,034 working adults	the United States	A^+^; I^+^; C^−^ with openness
Tokar and Swanson (1995)	Big 5 PTs	The NEO Five-Factor Inventory (Costa and McCrae, 1992)	679 employed adults	the United States	A^+^; I^+^; C^−^ with openness
Tokar et al. (1995)	Big 5 PTs	The NEO-PI (Costa and McCrae, 1985)	193 university students	the United States	A^+^; I^+^ with openness
Wiggins (1976)	Job satisfaction	The Job Satisfaction Blank (Hoppock, 1935)	110 teachers of the educable mentally disabled	the United States	I^+^; R^−^, C^−^
Wiggins et al. (1983)	Job satisfaction	the Job Satisfaction Blank (Hoppock, 1935)	247 teachers	the United States	I^+^; R^−^
Zhang (2008)	Big 5 PTs	the NEO-FFI (Costa and McCrae, 1992)	79 second-year university students	Hong Kong	A^+^ with openness; C^+^ with conscientiousness

*^FU^, Fully unexpected; ^PU^, Partially unexpected; MBTI, Myers-Briggs Type Indicator; N, Intuitive; S, Sensing; P, Perceiving; J, Judging; SDS, Self-Directed Search; SVSDS, Short-version Self-Directed Search; VPI, Vocational Preference Inventory; A, Artistic, I, Investigative, C, Conventional, R, Realistic; Big 5 PTs, Big Five personality traits; NEO-PI, NEO Personality Inventory; NEO-PI-R, NEO Personality Inventory-Revised; ^+^Positively associated with the attribute/outcome variable concerned; and ^−^Negatively associated with the attribute/outcome variable concerned*.
